# Transgenic Cry1Ac cotton does not affect the development and fecundity of *Chrysoperla carnea*

**DOI:** 10.1371/journal.pone.0214668

**Published:** 2019-04-05

**Authors:** Ruifeng Ding, Deying Ma, Ahtam Uwais, Dongmei Wang, Jian Liu, Yao Xu, Haobin Li, Haiqiang Li, Hongsheng Pan

**Affiliations:** 1 College of Agronomy, Xinjiang Agricultural University, Key Laboratory of the Pest Monitoring and Safety Control of Crops and Forests of Xinjiang Uygur Autonomous Region, Urumqi, Xinjiang PR China; 2 Institute of Plant Protection, Xinjiang Academy of Agricultural Sciences, Key Laboratory of Integrated Crop Pest Management in Northwestern Oasis, Ministry of Agriculture, Scientific Observation and Experimental Station for Crop Pests in Korla, Ministry of Agriculture, Urumqi, Xinjiang PR China; Northwest A&F University, CHINA

## Abstract

The development and fecundity of the predator *Chrysoperla carnea* Stephens (Neuroptera: Chrysopidae) were assessed by feeding *Aphis gossypii* Glover (Hemiptera: Aphididae) that had been reared on transgenic *Bacillus thuringiensis* (Bt) cotton SGK321 and a non-Bt cotton control (SY321) for two successive generations. We found no significant differences in the developmental stage duration, stage survival, or egg hatch rate between *C*. *carnea* fed *A*. *gossypii* reared on the Bt and non-Bt cotton. The fecundity per female over a 25-day observation period was very similar between treatments; for *C*. *carnea* fed *A*. *gossypii* reared on SGK321 vs. SY321, the amount of eggs laid was not significantly different in both generations. Furthermore, a population dynamics of *A*. *gossypii* and lacewing (mainly *C*. *carnea*) were highly similar in the SGK321 and SY321 treatments during 2016–2017. These results suggest that Bt cotton does not have a significantly negative or positive effect on *C*. *carnea* in terms of development, survival, fecundity, or population dynamics.

## Introduction

Transgenic cotton producing insecticidal proteins from the bacterium *Bacillus thuringiensis* (Bt) plays a significant role in insect pest management around the world. Bt cotton not only successfully controls several insect pests of cotton [e.g., *Helicoverpa armigera* (Hübner) (Lepidoptera: Noctuidae)] but also reduces pesticide use in other crops that previously required protection against target pests, thereby boosting crop yields and generating more income for farmers [1–3]. In China, Bt cotton has been used to control cotton bollworm since 1997, and it has already been adopted by 95% of farmers in northern China, where it plays a prominent role in pest control in cotton fields [4]. Transgenic Bt cotton has contributed substantially to the reduction of damage by cotton bollworm. Because of its widespread use, efforts to evaluate its safety have received a great deal of attention worldwide, and its potential for harmful effects on non-target species, especially natural predators [5], has been assessed. Widespread planting of Bt cotton has the potential to change the arthropod community in cotton [6], thus affecting populations of sucking pests, such as cotton aphids [7–8], mirid bugs [9], and others.

Cotton aphid *Aphis gossypii* (Hemiptera: Aphididae) is an important sucking pest of cotton worldwide that can affect cotton plants both by its direct feeding and through its ability to transmit diseases [10]. Predators in cotton fields, such as lacewings, ladybeetles are very important natural enemies in cotton fields, and can control the aphids effectively[11–12].

Lacewings (Chrysopidae) are important predators in cotton fields due to their high predation rates and ecological plasticity. Some widespread species are common in crop fields worldwide, including in China [13]. *Chrysoperla carnea* Stephens (Neuroptera: Chrysopidae) is an important natural predator that can be reared in the laboratory and used for pest control in the field [14]. This species preys on various pests in natural and cultivated fields, such as aphids, cotton bollworms, whiteflies, and the eggs and young larvae of Lepidoptera [15–19]. Green lacewings have also been used to assess the potential non-target effects of insecticides [20–21], fungicides [22], and GE plants [23–29] on the third trophic level in agricultural ecosystems. Overall, lacewings play an important role in biological control and integrated pest management in cotton fields.

The Xinjiang Uygur Autonomous Region (XJR) is the largest commercial cotton production region in China. The area of cotton cultivation in the region has expanded in recent years, reaching 2.15 million hectares in 2016 and accounting for more than 80% of all cotton production in China [30]. In Xinjiang, the semi-arid drought-prone climate characteristics are considerably different from that of other areas of China [31]. Therefore, the population dynamics of arthropods and composition of insect species in cotton fields in the XJR differ widely from that in the Yellow River Region or the Changjiang River Region [13].

Many studies have examined the direct and indirect effects of Bt on non-target species, including predators [5,32–34]. Most studies on the effects of Bt toxins on lacewings have focused on the direct effects using artificial diets, and these studies have demonstrated that green lacewing larvae are not sensitive to the toxins of Cry1Ab, Cry1Ac, or Cry2Aa [27,35]. Because reduced prey quality has been reported from tri-trophic studies with other predators, the effects of the Bt toxin on green lacewings may be indirectly mediated by lower prey quality, but only trace amounts of Cry proteins in aphids fed Bt crops can be tested[36]. Because *A*. *gossypii* may acquire Cry Bt toxin upon ingestion of cotton plant sap, it may then transmit the toxin to aphid predators. Initial studies of this interaction focused on the transfer of Bt toxin to predators, and lacewing larvae have been commonly examined in tri-trophic experiments through their predation activity [37–38]. The mite *Tetranychus urticae* (Koch) and Bt-resistant larvae of *H*. *armigera* were used as prey and Bt as the toxin, and no adverse effects on green lacewing were observed [24,34,39]. This study assessed the potential for impacts of Bt cotton on the predator *C*. *carnea* when feeding on *A*. *gossypii* reared on Bt vs. non-Bt cotton for two generations, and we also monitored their population dynamics under field conditions during 2016–2017. The results are available for identifying the ecological effect of Bt cotton on predatory lacewing.

## Materials and methods

### Cotton cultivation and management

Seeds of SGK321 containing the Cry1Ac gene and the non Bt variety ShiYuan321 (SY321) (the parental cultivar of SGK321) were both provided by the Institute of Plant Protection, Chinese Academy of Agricultural Sciences. The study site was at the Scientific Observation and Experimental Station for Crop Pests in Korla, XJR (85°48′N, 41°44′S). Two cotton varieties were planted on 10 April 2016 and 8 April 2017 in plots using drip irrigation under film technology. The planting density was 180000 plants/hm^2^ and plot size was 2000 m^2^ for each cotton variety, and three plots were established per variety, the space between the two treatment plots was 2 m, and all the plots were randomly arranged random arrangement in the fields. All cotton plots were not treated with any chemical pesticides for this study, other management practices followed normal agronomic practices for the region.

### Development and fecundity in the laboratory

#### Predator rearing

*Chrysoperla carnea* individuals were collected in a commercial cotton field in 2012, and adults and larvae were fed 20% honey water and eggs of *Sitotroga cerealella* (Olivier)[40] at the insectarium of the institute of plant protection, Xinjiang Academy of Agricultural Sciences, under constant conditions for adults oviposition (temperature at 23±1°C, relative humidity at 65%±5%, and L/D cycle of18/6 h).

This experiment began on June 2017 once the *A*. *gossypii* populations of the experimental plots were sufficient for the study. Two generations of *C*. *carnea* were reared on *Aphis gossypii* collected from either the SGK321 or SY321 cotton varieties. Experiments with the first and second predator generations began on 10 June and 8 July, 2017, respectively, and under constant conditions (temperature at 26±1°C, relative humidity at 65%±5%, and L/D cycle of18/6 h).

#### Lacewing larval development and survival

*C*. *carnea* neonate larvae (<8 h) were collected from containers and reared individually in plastic culture dishes (9 cm in diameter, 1.5 cm high). Neonate larvae were fed *A*. *gossypii* on cotton leaves picked from either SGK321 or SY321 cotton plants. Predators in their 1^st^, 2^nd^ and 3^rd^ instar stages were provided with a minimum of 50, 100, and 300 aphids as prey and leaves with *A*. *gossypii* were replaced every 24 h. The experiment had three replicates for each treatment, with 30 first instar larvae in each replication. The stage duration of each larval instar and the pupation period was calculated from observations of the numbers of live or dead lacewings until adult emergence, and observed every 8 hours.

#### Adult fecundity and hatch rate

Twenty newly emerged *C*. *carnea* pairs developed on Bt cotton and non Bt cotton were selected respectively, and each pair was reared in a plastic pot (20 cm in diameter, 30 cm high) with honey water (20%) for food.Cauze (3 cm width, 20 cm length) were hung inside cages for female lacewings to lay eggs on. Gauze and a rubber band were used to seal the pots. The gauze was removed daily to record the number of eggs laid by each female and then replaced with fresh gauze. Put a wet cotton balls(d = 2 cm) for keep moist and changed it daily. The fecundity experiment lasted 25 days, and each day the number of hatched eggs and dead adults were recorded. After obtaining new adults of *C*. *carnea*, the experiment was repeated with the second generation of lacewings.

### Population dynamics in field plots

According to a previous survey, three species of lacewings were identified in local cotton fields, i.e., *C*. *carnea*, *C*. *formosa Brauer* and *C*. *sinica* Tjeder [41–42], and *C*. *carnea* was the dominant species. These plots were investigated once every 7 d from 1 June to 17 August in both years, the average plant height were 30cm in 1 June to 100cm in 17August, and average of leaves were sampled. In each plot, a total of 5 points were randomly chosen in which 20 plants for visual sampling. On each plant, the numbers of *A*. *gossypii* and lacewings (larva and adults) were counted [43]. Instars and adults of lacewings were recorded respectively, but combined statistics were performed during analysis.

### ELISA for Bt cotton

To confirm that SGK321 expressed the Cry1Ac protein and the control variety did not, one leaf was collected from the upper portion of five randomly selected plants from each plot (cotton variety) in 2017. Leaf sampling was repeated every 10 days from 10 June to 8 September, and *A*. *gossypii* or *C*. *carnea* can be found in both and control cotton fields. Leaves were stored at -20°C. ELISA testing was used for the detection of the Bt protein. A Cry1Ac kit (EnviroLogix) was used for the ELISA tests, which followed the manufacturer’s instructions. Sample concentrations of Bt toxin (absorption values of 450 nm) were measured against a standard curve of protein standard absorption values of 0, 1, 2, and 4 ng/g.

### Data analysis

The datasets on the development time, preoviposition period, pupal period, and number of eggs per female were all log-transformed for normality, and the percentage data sets (survival and hatch rate) were arcsine-transformed. The egg number data were using original data. Larval development was analysed using 90 data points (3×30/replicate) for each treatment. Survival data were analysed based on means of three replicates (30 individuals per replicate). The fecundity and hatch rate datasets were analysed using 20 data points for each treatment; and the fecundity data were adjusted according to the number of dead females for analysis because they could not survive for 25 days. The population dynamics of *A*. *gossypii* and lacewings between SGK321 and SY321 were analyzed respectively by t-test for each year. All analyses were performed using SPSS 19.0 software [44].

## Results

### Cry1Ac protein expression for Bt cotton leaves

**Fig 1:**
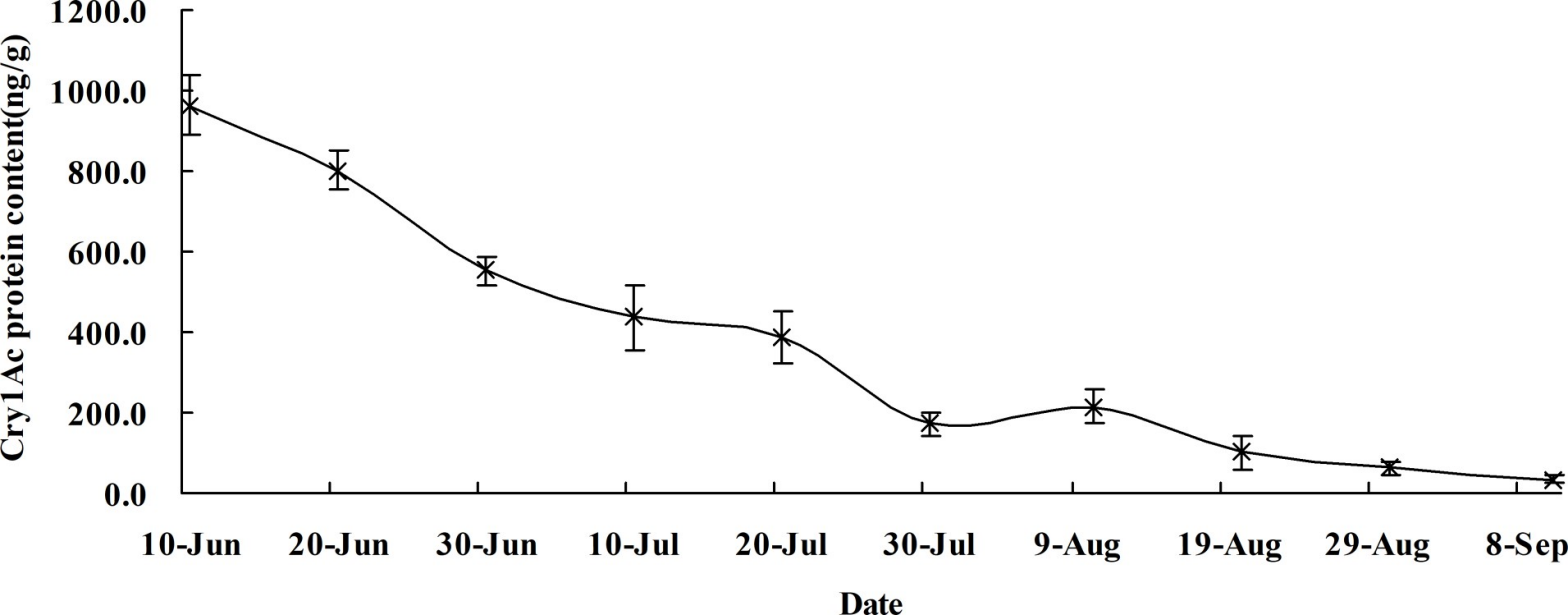
Cry1Ac protein content of cotton leaves during the cotton production season (ng/g).

Cry1Ac protein of fresh weight (FW) expression in the leaves of SGK321 detected by ELISA was 964.2 ng/g on June 10, and it dropped to 802.7 ng/g on June 20 in 2017. The decrease continued until September, with a minimum content of 35.1 ng/g on September 10 (Fig 1). No Cry1Ac protein was detected in the SY321 leaves.

### Effect of Cry1Ac cotton aphids on the developmental duration of *C*. *carnea* larvae

The durations of the 1^st^, 2^nd^ and 3^rd^ instars, the total larval stage for the first and second predator generation in the experiment were no significant difference between fed *A*. *gossypii* reared on SGK321 (Bt) and SY321(non-Bt) cotton. Similarly, the preoviposition period the pre-pupal stage, and the pupae for the first and second generation between two treatments were not significant (Table 1).

**Table 1:** Duration of the developmental stages of two generation of *Chrysoperla carnea* fed *Aphis gossypii* reared on SGK321 (Bt) or SY321 (control) cotton.

Developmental period		Cotton variety		t-test		
		SGK321	SY321	t	df	p
1^st^ instar	1^st^ generation	67.9±0.58	67.2±0.66	0.714	163	0.476
	2^nd^ generation	66.7±0.79	67.2±0.71	-0.509	165	0.611
2^nd^ instar	1^st^ generation	72.3±0.76	72.2±0.67	0.101	154	0.920
	2^nd^ generation	73.1±0.80	73.5±0.61	-0.386	157	0.700
3^rd^ instar	1^st^ generation	95.6±0.76	95.8±0.63	-0.213	147	0.832
	2^nd^ generation	94.7±0.65	95.2±0.79	-0.420	151	0.675
Larval phase	1^st^ generation	235.7±1.25	235.5±1.10	0.165	147	0.869
	2^nd^ generation	234.8±1.18	235.8±1.11	-0.619	151	0.537
Pre-pupal stage	1^st^ generation	42.8±0.60	43.0±0.73	-0.163	139	0.871
	2^nd^ generation	42.3±0.77	42.4±0.67	-0.044	144	0.965
Pupal stage	1^st^ generation	158.8±0.83	158.9±0.75	-0.090	135	0.928
	2^nd^ generation	160.5±0.78	159.8±0.72	0.639	140	0.524
Preoviposition period	1^st^ generation	91.8±0.49	91.6±0.50	0.360	130	0.720
	2^nd^ generation	92.8±0.48	92.1±0.48	0.944	137	0.347

Data are presented as mean ± SE. P values are for the comparison between the treatment and control.

### Effect of Cry1Ac cotton on the survival- survival of *C*. *carnea*

The stage survival of *C*. *carnea* fed *A*. *gossypii* reared on the two cotton varieties was not different between lacewings fed on aphids reared on Bt vs control cotton, and this finding was true for both the 1^st^ and 2^nd^ lacewing generations, suggesting that there was no significant effect at this stage. The survival of the 1^st^ instar and the pre-pupal stage of *C*. *carnea* fed *A*. *gossypii* reared on SGK321 (Bt) was lower than *C*. *carnea* fed *A*. *gossypii* reared on SY321 (control) cotton in both the 1^st^ and 2^nd^ lacewing generations, whereas the 2^nd^ instar and pupal stage’s survival and the preoviposition period of *C*. *carnea* fed *A*. *gossypii* reared on SGK321 (Bt) were longer than those of *C*. *carnea* fed *A*. *gossypii* reared on SY321 (control) cotton in both the 1^st^ and 2^nd^ generations. The survival of the total larval stage of *C*. *carnea* fed *A*. *gossypii* reared on SGK321 (Bt) was higher than that of *C*. *carnea* fed *A*. *gossypii* reared on the SY321 (control) in the 1^st^ generation, although the opposite result was observed in the 2^nd^ instar (Table 2),however, there was no significant difference between two varieties, and reversed results between two generation may caused by experimental error.

**Table 2:** Survival of the immature stages of two successive generations of *Chrysoperla carnea* fed *Aphis gossypii* reared on SGK321 (Bt) verses SY321 (control) cotton varieties.

Survival (%)		Cotton variety		t-test		
		SGK321	SY321	t	df	p
1^st^ instar	1^st^ generation	91.1±1.10	92.2±4.00	-0.273	4	0.798
	2^nd^ generation	92.2±1.10	93.3±1.93	-0.509	4	0.637
2^nd^ instar	1^st^ generation	95.2±2.42	94.0±1.20	0.444	4	0.680
	2^nd^ generation	95.3±2.37	95.2±1.30	0.025	4	0.981
3^rd^ instar	1^st^ generation	96.3±2.14	95.0±1.04	0.547	4	0.613
	2^nd^ generation	96.2±2.22	96.1±2.31	0.021	4	0.984
Larval phase	1^st^ generation	83.3±0.00	82.2±2.94	0.363	4	0.735
	2^nd^ generation	84.5±2.23	85.6±4.43	-0.222	4	0.835
Pre-pupal stage	1^st^ generation	94.7±1.33	94.8±2.62	-0.034	4	0.974
	2^nd^ generation	94.8±1.24	95.9±2.52	-0.392	4	0.715
Pupal stage	1^st^ generation	97.2±1.40	97.2±2.45	0.017	4	0.987
	2^nd^ generation	97.2±1.39	97.2±1.45	0.033	4	0.975
Preoviposition period	1^st^ generation	97.1±1.43	95.6±0.07	1.045	4	0.355
	2^nd^ generation	98.6±1.40	97.1±1.51	0.745	4	0.497

Data are mean ± SE. Temperature was 23 ± 2°C, RH was 65 ± 5% and L/D cycle was 18/6 h.

### Effect of Cry1Ac cotton aphids on the fecundity of *C*. *carnea*

For the 1^st^ generations, the total fecundity per female over 25 days was (316±21.1) eggs for *C*. *carnea* fed *A*. *gossypii* reared on SGK321 (Bt) and (313±18.3)eggs for *C*. *carnea* fed *A*. *gossypii* reared on SY321 (control). For the 2^nd^ generation, the total fecundity values were (315±19.8) and (334±23.6) for these two treatments, and significant differences were not observed (1^st^ generation: t = 0.123, df = 32, p = 0.903; 2^nd^ generation: t = -0.602, df = 31, p = 0.551).

The hatch rate of eggs from *C*. *carnea* fed *A*. *gossypii* reared on SGK321 (Bt) was (91.6±0.55)% in the 1^st^ generation, whereas that of eggs of *C*. *carnea* fed *A*. *gossypii* reared on SY321 (control) was (91.8±0.52)%. The hatch rates for the 2^nd^ generation of these treatments were (92.1±0.68)% and (91.1±1.15)%, and significant differences were not observed between the two treatments (1^st^ generation: t = -0.734, df = 32, p = 0.734; 2^nd^ generation: t = 792, df = 31, p = 0.434).

### Field dynamics of *Aphis gossypii* and lacewing (mainly *C*. *carnea*)

**Fig 2:**
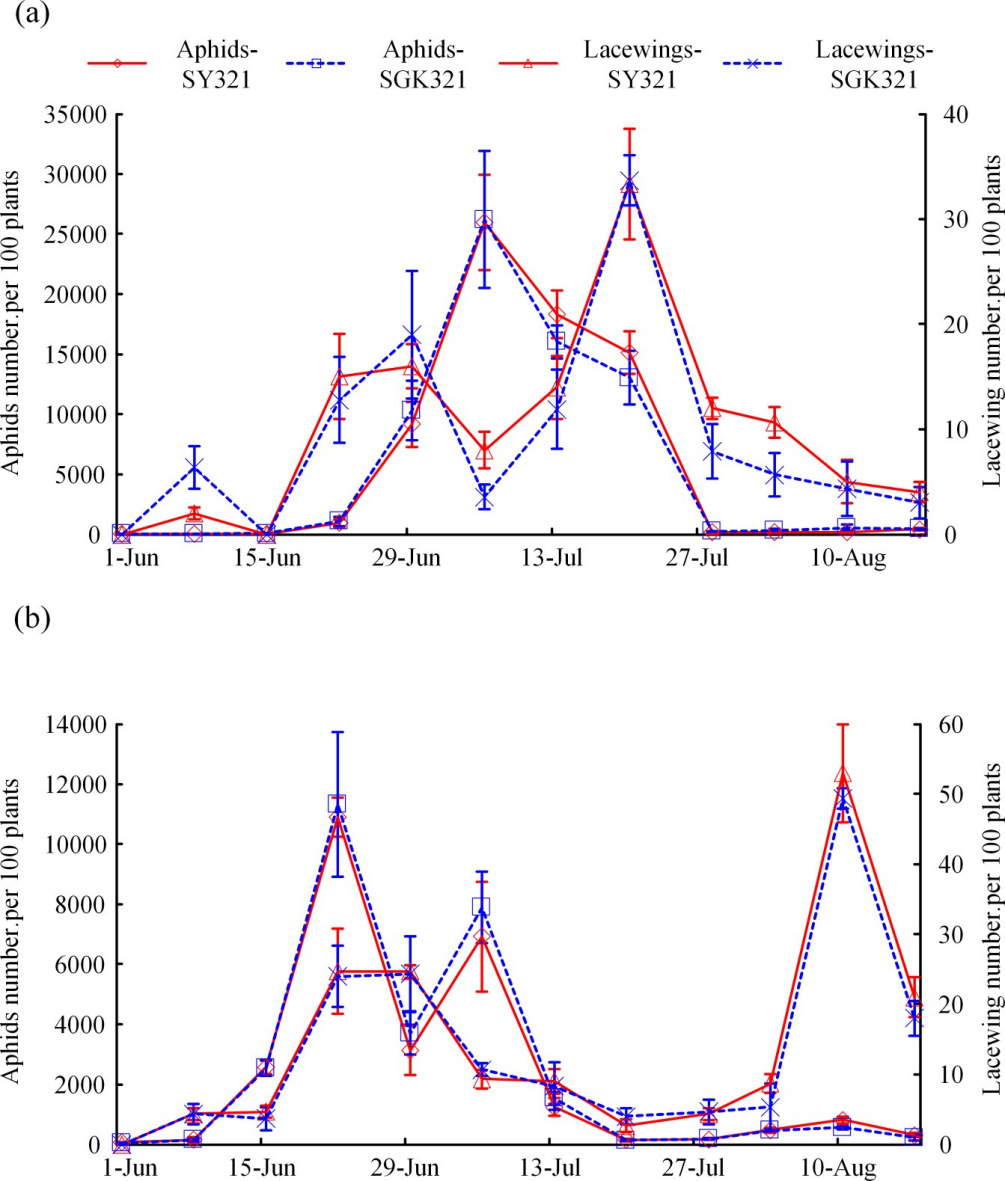
Population dynamics of *Aphis gossypii* and lacewings (mainly *Chrysoperla carnea*) in SY321 and SGK321 in 2016 (a) and 2017 (b).

The field dynamics of *Aphis gossypii* and lacewings were investigated in SY321 and SGK321 during 2016–2017. The number of *Aphis gossypii* and lacewings were similar in SY321 and SGK321 cotton fields in each year, and no significant difference have been found between the transgenic Bt cotton and non-transgenic cotton for each pest and predator group in each year (Fig 2 and Table 3).

**Table 3:** The T-test on the number of lacewings and aphids in Bt and non-Bt cotton respectively. Table 3. The T-test on the number of lacewings and aphids in Bt and non-Bt cotton respectively.

Years	Date	T-test of Lacewings in Bt and non-Bt cotton fields			T-test of Aphids in Bt and non-Bt cotton fields		
		t	df	P	t	df	P
2016	June 1	—	—	—	0.295	4	0.783
2017		—	4	—	0.894	4	0.422
2016	June 8	-2.055	4	0.109	0.491	4	0.649
2017		0.000	4	1.000	0.019	4	0.986
2016	June 15	—	—	—	-0.789	4	0.474
2017		0.530	4	0.624	0.116	4	0.913
2016	June 22	0.408	4	0.704	-0.259	4	0.808
2017		0.089	4	0.933	-0.168	4	0.875
2016	June 29	-0.467	4	0.665	-0.359	4	0.738
2017		0.061	4	0.954	-0.525	4	0.627
2016	July 6	2.055	4	0.109	-0.033	4	0.975
2017		-0.834	4	0.451	-0.477	4	0.678
2016	July 13	0.414	4	0.700	0.948	4	0.397
2017		0.175	4	0.869	-0.859	4	0.439
2016	July 20	-0.058	4	0.957	0.746	4	0.497
2017		-0.918	4	0.411	0.147	4	0.890
2016	July 27	1.414	4	0.230	-0.622	4	0.568
2017		-0.169	4	0.874	-0.597	4	0.583
2016	August 3	2.004	4	0.116	-1.178	4	0.304
2017		0.928	4	0.406	0.111	4	0.917
2016	August 10	0.200	4	0.851	-1.562	4	0.193
2017		0.513	4	0.635	1.996	4	0.117
2016	August 17	0.548	4	0.613	-0.537	4	0.620
2017		0.783	4	0.477	0.684	4	0.532

Data are mean ± SE.

## Discussion

Safety evaluations of Bt cotton must include assessments of both direct (Cry protein-related) and indirect effect. In this study, we found no influence of Bt cotton on the larval lacewing parameters or female fecundity of *C*. *carnea* in either of two successive generations. The developmental times of *C*. *carnea* life stages appeared normal for all three larval instars (i.e., the pre-pupal and pupal stages and preoviposition period), which is similar to the results of a previous study [45]. These results suggest that *C*. *carnea* was not negatively affected by Bt cotton.

The approach to assessing the potential impact of GE plants on non-target organisms requires the evaluation of the level of insecticidal protein expression of the plant [46–47]. In this study, we measured the protein content of the Bt toxin in Bt cotton leaves and found that Bt cotton expressed the Bt toxin normally, although the levels of Bt proteins in the tender leaves of SGK321 decreased gradually with increasing age of the Bt cotton, and some results showed Cry-protein concentrations strongly decreased with increasing trophic level to values mostly below the detection limit in predators[48]. Therefore, the Bt toxin may not be actually ingested by *A*. *gossypii*,and it is unlikely that the aphids contain significant amounts of Cry protein[36, 49–50] This failure of Bt to be present in aphid diets has been observed with different aphid species reared on Bt maize. Although Bt maize expresses higher levels of Bt toxin than cotton, little or no Bt toxin was detected in aphids fed on Bt corn[51–53], and a new study showing only traces of some Cry protein contained in aphids fed Bt maize[36, 54]. The above findings can be attributed to the fact that aphids cannot ingest considerable amounts of Bt proteins when fed transgenic Bt crops because they feed solely on the phloem of the plants, and phloem has a very low content of Bt toxin[39]. There is evidence that aphid samples from Bt crops containing relative high amounts of Cry proteins might have been caused by contamination for example thrips or thrips faces [36].

Few studies have focused on the arthropod diversity, communities and population dynamics of Bt cotton fields in the XJR [42,55–57], most investigations over two or three years and finding no negative effects because one-year field research may not provide strong evidence in the ecological assessments of transgenic Bt cotton. Diverse ecological factors change greatly over time and can affect the growth of crops and populations of pest and natural enemies [58]. Investigations over two consecutive years were carried out in this study because the population dynamics of *A*. *gossypii* and *C*. *carnea* changed considerably over this time period but maintained the same trend both on transgenic Bt cotton and non-Bt cotton in the same year, which indicated that different trends in the population dynamics of pests and predators were greatly affected by annual factors but not by the Bt gene. A combination of bioassays in the laboratory indicated that the transgenic Bt cotton did not produce adverse effects on the predator, and this result was similar to that of previous studies [32–35].

Many studies have shown that Bt toxins have no direct negative effects on the larvae of green lacewings [23, 59] and the larvae of C. *sinica* are not sensitive to Bt [35]. We collected aphids in cotton fields to better simulate the field situations for predation and found results very similar to those of previous research. Moreover, the population dynamics of *A*. *gossypii* and lacewings (mainly *C*. *carnea*) fed on Bt cotton and non-Bt cotton during 2016–2017 indicated that transgenic Bt cotton did not affect individual development and fecundity or the population dynamics of C. *carnea*.

## References

[1] Shelton AM., Zhao JZ, Roush RT. Economic, ecological, food, safety, and social consequences of the deployment of Bt transgenic plants. Annual Review of Entomology. 2002; 47: 845–881. 10.1146/annurev.ento.47.091201.145309 11729093

[2] Carrière Y, Ellers-Krik C, Sisterson M, Antilla L, Whitlow M, Dennehy T J, et al Long-term regional suppression of pink bollworm by *Bacillus thuringiensis* cotton. Proceedings of the National Academy of Sciences. 2003; 100(4): 1519–1523. 10.1073/pnas.0436708100 PMC149864 12571355

[3] Wu KM, Lu YH, Feng HQ, Jiang YY, Zhao JZ. Suppression of cotton bollworm in multiple crops in China in areas with Bt toxin-containing cotton. Science. 2008; 321: 1676–1678. 10.1126/science.1160550 18801998

[4] Lu YH, WU KM, Jiang YY, Xia B, Li P, Feng HQ, et al Mirid bug outbreaks in multiple crops correlated with wide-scale adoption of Bt cotton in China. Science. 2010; 328(5982): 1151–1154. 10.1126/science.1187881 20466880

[5] Romeis J, Naranjo SE, Meissle M, Shelton AM. Genetically engineered crops help support conservation biological control. Biological Control, 2019; 130: 136–154.

[6] Torres JB, Ruberson JR. Canopy- and ground dwelling predatory arthropods in commercial Bt and non-Bt cotton Telds: patterns and mechanisms. Environmental Entomology. 2005; 34(5): 1242–1256.

[7] Deng SD, Xu J, Zhang QW, Zhou SW, Xu GJ. Effect of transgenic Bt cotton on population dynamics of the non-target pests and natural enemies of pests. Acta Entomologica Sinica. 2003; 46(1):1–5.

[8] Men XY, Ge F, Liu XH, Yardim EN. Diversity of arthropod communities in transgenic Bt cotton and nontransgenic cotton agroecosystems. Environmental Entomology. 2003; 32(2): 270–275.

[9] Wu KM., Li WD, Feng HQ, Guo YY. Seasonal abundance of the mirids, *Lygus lucorum* and *Adelphocoris spp*. (Hemiptera: Miridae), on Bt cotton in northern China. Crop Protect. 2002; 21(10): 997–1002.

[10] Reddall A, Ali A, Able JA, Tesoriero L, Wright PR, Rezaian MA, et al Cotton bunchy top: an aphid and graft transmitted cotton disease. Australasian Plant Pathology. 2004; 33(2): 197–202.

[11] Sisterson MS, Biggs RW, Olson C, Carrière Y, Dennehy T J, Tabashnik BE. Arthropod abundance and diversity in Bt and non-Bt cotton fields. Environmental Entomology. 2004; 33(4): 921–929.

[12] Mellet MA, Schoeman AS. Effect of Bt-cotton on Chrysopids, ladybird beetles and their prey: Aphids and whiteflies. Indian Journal of Experimental Biology. 2007; 45(6): 554–562. 17585692

[13] Wu KM, Guo YY. The evolution of cotton pest management practices in China. Annual Review of Entomology. 2005; 50(1): 31–52. 10.1146/annurev.ento.50.071803.130349 15355239

[14] Syed AN, Ashfaq M, Ahmad S. Comparative Effect of Various Diets on Development of *Chrysoperla carnea* (Neuroptera: Chrysopidae). International Journal of Agriculture & Biology. 2008; 10(6): 728–730.

[15] Balasubramani V, Swamiappan M. Development and feeding potential of green lacewing *Chrysoperla carnea* Steph. (Neur. Chrysopidae) on different insect pests of cotton. Anzeiger Für Schädingskunde, Pflanzenschutz Umweltschutz. 1994; 67(8): 165–167.

[16] Senior LJ, McEwen PK. Laboratory study of *Chrysoperla carnea* (Stephens) predation on *Trialeurodes vaporariorum* (Westwood) (Hom., Aleyrodidae). Journal of Applied Entomology. 1998; 122(1–5): 99–101.

[17] Easterbrooka MA, Fitzgeralda JD, Solomona MG. Suppression of aphids on strawberry by Augmentative releases of larvae of the lacewing *Chrysoperla carnea* (Stephens), Biocontrol Science & Technology. 2006; 16(9):893–900.

[18] Zia K, Hafeez F, Khan RR, Arshad M, Naeem-Ullah U. Effectiveness of *Chrysoperla carnea*(Stephens) (Neuroptera: Chrysopidae) on the population of *Bemisia tabaci* (Homoptera: Aleyrodidae) in different cotton genotypes. Journal of Agriculture & Social Sciences. 2008; 4(3): 112–116.

[19] Bahar MH, Stanley JN, Gregg PC, Del Socorro AP, Kristiansen P. Comparing the predatory performance of green lacewing on cotton bollworm on conventional and Bt cotton. Journal of Applied Entomology. 2011; 136(4): 263–270.

[20] Romeis J, Meissle M, Naranjo SE, Li YH, Bigler F. Frontiers in Plant Science. 2014; 391(5): 1–10. 10.3389/fpls.2014.00391 PMC4129496 25161661

[21] Sabry KH, Ei-sayed AA. Biosafety of a biopesticide and some pesticidesused on cotton crop against green lacewing, *Chrysoperla carnea* (Stehens) (Neuroptera: Chrysopidae). Journal of Biopesticides. 2011; 4(2): 214–218.

[22] Nasreen A, Cheema G.M, Iqbal M. Relative toxicity of different fungicides against larvae of green lacewing, Chrysoperla carnea (Neuroptera: Chrysopidae). South Pacific Studies. 2005; 26(1): 7–13.

[23] Romeis J, Dutton A, Bigler F. *Bacillus thuringiensis toxin* (Cry1Ab)has no direct effect on larvae of the green lacewing *Chrysoperla carnea* (Stephens) (Neuroptera: Chrysopidae). Journal of Insect Physiology. 2004; 50(2–3): 175–183. 10.1016/j.jinsphys.2003.11.004 15019519

[24] Obrist LB, Dutton A, Albajes R, Bigler F. Exposure of arthropod predators to Cry1Ab toxin in Bt maize fields. Ecological Entomology. 2006; 31(2): 143–154.

[25] Li YH, Meissle M, Romeis J. Consumption of Bt Maize Pollen Expressing Cry1Ab or Cry3Bb1 Does Not Harm Adult Green Lacewings, *Chrysoperla carnea* (Neuroptera: Chrysopidae). PLoS ONE. 2008; 3(8): 1–8. 10.1371/journal.pone.0002909 PMC2488376 18682800

[26] Li YH, Wang Y Y, Romeis J, Liu QS, Chen XP, Peng YF. Bt rice expressing Cry2Aa does not cause direct detrimental effects on larvae of *Chrysoperla sinica*. Ecotoxicology. 2013; 22(9): 1413–1421. 10.1007/s10646-013-1127-0 24057602

[27] Li YH, Hu L, Romeis J, Wang J, Han LZ, Chen XP, et al Use of an artificial diet system to study the toxicity of gut-active insecticidal compounds on larvae of the green lacewing *Chrysoperla sinica*. Biological Control. 2014; 69: 45–51.

[28] Wang YY, Li YH, Romeis J, Chen XP, Zhang J, Chen HY, et al Consumption of Bt rice pollen expressing Cry2Aa does not cause adverse effects on adult *Chrysoperla sinica* Tjeder (Neuroptera: Chrysopidae). Biological Control. 2012; 61(3): 246–251.

[29] Tian JC, Wang XP, Long LP, Romeis J, Naranjo SE, Hellmich RL, et al Bt crops producing Cry1Ac, Cry2Ab, and Cry1F do not harm the green lacewing, *Chrysoperla rufilabris*. PLoS ONE. 2013; 8(3): e60125 10.1371/journal.pone.0060125 23544126 PMC3609736

[30] Statistics Bureau of Xinjiang Uygur Autonomous Region. Xinjiang statistical yearbook China Statistics Press 2017.

[31] National Bureau of Statistics of China. China statistical yearbook China Statistics Press 2016

[32] Romeis J, Meissle M, Bigler F. Transgenic crops expressing Bacillus thuringiensis toxins and biological control. Nature Biotechnology. 2006; 24(1): 63–71. 10.1038/nbt1180 16404399

[33] Marvier M, Mccreedy C, Regetz J, Kareiva P. A meta-analysis of effects of Bt cotton and maize on nontarget invertebrates. Science. 2007; 316: 1475–1477. 10.1126/science.1139208 17556584

[34] Wolfenbarger LL, Naranjo SE, Lundgren JG, Bitzer RJ, Watrud LS. Bt crop effects on functional guilds of nontarget arthropods: a meta-analysis. PLoS ONE. 2008; 3(5): e2118 10.1371/journal.pone.0002118 18461164 PMC2346550

[35] Li YH, Chen XP, Hu L, Romeis J, Peng YF. Bt rice producing Cry1C protein does not have direct detrimental effects on the green lacewing *Chrysoperla sinica*(Tieder). Environmental Toxicology and Chemistry. 2014; 33(6): 1391–1397. 10.1002/etc.2567 24619941

[36] Lawo NC, Wäckers FL, Romeis J. Characterizing indirect prey-quality mediated effects of a Bt crop on predatory larvae of the green lacewing, *Chrysoperla carnea*. Journal of Insect physiology. 2010; 56(11): 1702–1710. 10.1016/j.jinsphys.2010.06.012 20619267

[37] Dutton A, Klein H, Romeis J, Bigler F. Uptake of Bt-toxin by herbivores feeding on transgenic maize and consequences for the predator *Chrysoperla carnea*. Ecological. Entomolology. 2002; 27(4): 441–447.

[38] Li YH, Meissle M, Romeis J. Use of maize pollen by adult *Chrysoperla carnea*(Neuroptera: Chrysopidae) and fate of Cry proteins in *Bt*-transgenic varieties. Journal of Insect Physiology. 2010; 56(2): 157–164. 10.1016/j.jinsphys.2009.09.011 19782688

[39] Romeis J, Meissle M. Non target risk assessment of Bt crops cry protein uptake by aphids. Journal of Applied Entomology. 2010; 135(1–2): 1–6.

[40] Bao JZ, Gu DX. Biologic control in China Taiyuan: Shanxi Science and Technology Publishing House 1998; 203–224.

[41] Ding RF, Maimaiti Y, Yang HL, Liu J, Wang DM, Akedan W, et al Development and fecundity of *Chrysoperla carnea* reared on *Aphis gossypii* on transgenic Bt±CpTI cotton. Chinese Journal of Applied Entomology. 2014; 51(1): 178–184.

[42] Mamat Y, Ahtam U, Yasin S, LIU J, Arziguli R, Ding RF. Growth, development and adult fecundity of *Chrysoperla carnea* feeding on different larval diets. Plant protection. 2015; 41(4): 35–38.

[43] Xu Y, Wu KM, Li HB, Liu J, Ding RF, Wang F, et al Effects of transgenic Bt+CpTI cotton on field abundance of non-target pests and predators in Xinjiang, China. Journal of Integrative Agriculture. 2012; 11(9): 1493–1499.

[44] Coakes SJ. SPSS: analysis without anguish: version 20.0 for Windows John Wiley & Sons 2013.

[45] Guo JY, Wan FH, Dong L, LÖvei GL, Han ZJ. Tritrofic interactions between *Bt* cotton plants, the aphids *Aphis gossypii* Glover,1827(Hemiptera: Aphidiae), and the predator, *Chrysoperla externa* (Hagen, 1861)(Neuroptera: Chrysopidae). Enviromental Entomology 2008; 37(1): 263–270. 10.1603/0046-225x(2008)37[263:tibbct]2.0.co;2 18348819

[46] Romeis J, Bartsch D, Bigler F, Candolfi MP, Gielkens M M.C, Hartley SE, et al Assessment of risk of insect-resistant transgenic crops to nontarget arthropods. Nuture Biotechnology. 2008; 26(2): 203–208. 10.1038/nbt1381 18259178

[47] Andow DA, Zwahlen C. Assessing environmental risks of transgenic plants. Ecology Letter. 2010; 9(2): 196–214. 10.1111/j.1461-0248.2005.00846.x 16958885

[48] Eisenring M, Romeis J, Naranjo SE, Meissle M. Multitrophic Cry-protein flow in a dual-gene Bt-cotton field. Agriculture, Ecosystems and Enviroment. 2017; 247: 283–289.

[49] Lawo NC, Wäckers FL, Romeis J. Indian Bt cotton varieties do not affect the performance of cotton aphids. PLoS ONE, 2009; 4(3): e4804 10.1371/journal.pone.0004804 19279684 PMC2653191

[50] Meissle M, Romeis J. Transfer of Cry1Ac and Cry2A proteins from genetically engineered Bt cotton to herbivores and predators. Insect Science, 2018; 25: 823–832. 10.1111/1744-7917.12468 28374515

[51] Raps A, Kehr J, Gugerli P, Moar WJ, Bigler F, Hilbeck A. Immunological analysis of phloem sap of *Bacillus thuringiensis* corn and of the non-target herbivore *Rhopalosiphumpadi* (Homoptera: Aphididae) for the presence of Cry1Ab. Molecular Ecology. 2001; 10(2): 525–533. 11298965 10.1046/j.1365-294x.2001.01236.x

[52] Head G, Brown CR, Groth ME, Duan JJ. Cry1Ab protein levels in phytophagous insects feeding on transgenic corn: implications for secondary exposure risk assessment. Entomologia Experimentalis et Applicata. 2001; 99(1): 37–45.

[53] Lundgren JG, Wiedenmann RN. Tritrophic interactions among Bt (Cry3Bb1) corn, aphid prey, and the predator *Coleomegilla maculata* (Coleoptera: Coccinellidae). Environmental Entomology. 2005; 34(6): 1621–1625.

[54] Svobodová Z, Shu Y, Habuštová OS, Romeis J, Meissle M. Stacked Bt maize and arthropod predators–Exposure to insecticidal Cry proteins and potential hazards. Proceedings of the Royal Society B: Biological Science, 2017; 284: 20170440. 10.1098/rspb.2017.0440 PMC5543214 28724730

[55] Xu Y, WU KM, Li HB, Wang F, Sun SL, Li XY. Effect of transgenic Bt cotton on main pests and community of nature enemies in cotton fields. Xinjiang Agricultural Sciences. 2004; 41(5): 345–347.

[56] Li HB, Wu KM, Yang XR, Xu Y, Yao J, Wang F, et al Trend of occurrence of cotton bollworm and control efficacy of Bt cotton in cotton planting region of southern Xinjiang. Scientia Agricultura Sinica. 2006; 39(1): 199–205.

[57] Wang J, Lü ZZ, Men JW, He WJ, Jia YH. Effects of transgenic cotton on the population of major pests and natural enemies in Xinjiang cotton field. Xinjiang Agricultural Sciences. 2008; 45(3): 433–437.

[58] Romeis J, Shelton AM, Kennedy GG. Integration of insect-resistant genetically modified crops within IPM programs Spring Netherlands 2008; 5: 1–26.

[59] Ali I, Zhang S, Muhammd MS, Iqbal M, Cui JJ. Bt proteins have no detrimental effects on larvae of the green lacewing,*Chrysopa pallens* (Rambur) (Neuroptera: Chrysopidae). Neotropical Entomology. 2018; 47: 832–836. 10.1007/s13744-017-0526-y 28451986

